# Screening for Frailty in Older Emergency Patients and Association with Outcome

**DOI:** 10.3390/geriatrics5010020

**Published:** 2020-03-19

**Authors:** Siobhan Lewis, Louis Evans, Timothy Rainer, Jonathan Hewitt

**Affiliations:** 1 Department of Medicine, University Hospital of Wales, Heath Park, Cardiff CF14 4XW, UK; siobhanbrown@doctors.org.uk (S.L.); thrainer@cuhk.edu.hk (T.R.); 2 Department of Population Medicine, Cardiff University, Cardiff CF10 3XQ, UK; hewittj2@cardiff.ac.uk; 3 Department of Geriatric Medicine, 3rd Floor Academic Centre, Llandough Hospital, Penlan Road, Cardiff CF642XX, UK

**Keywords:** asthenia, emergencies, frailty, geriatrics

## Abstract

Older people have a high incidence of adverse outcomes after urgent care presentation. Identifying high-risk older patients early is key to targeting interventions at those patients most likely to benefit. This study used the Frailsafe three-point screening questions amongst older Emergency Department (ED) attendees. Consecutive unplanned ED attendances in patients aged ≥75 were assessed for Frailsafe status. The primary outcome was mortality at 180 days. A Frailsafe screen was completed in 356 patients, of whom 194/356 (54.5%) were Frailsafe positive. The mean age was 85.8 for Frailsafe screen positive and 82.2 for Frailsafe screen negative patients (*p* < 0.001). A positive Frailsafe screen was a predictor of death within 180 days of presentation to the ED and remained so after adjustment (AOR = 3.23, 95% CI 1.45–7.19, *p* = 0.004). A positive Frailsafe screen was an independent predictor of a new care home admission at 180 days (AOR = 8.95, 95% CI 2.01–39.83, *p* = 0.004). A positive Frailsafe screen was also predictive of a number of secondary outcomes, such as length of stay of >28 days (AOR 3.42, 95% CI 1.41–8.31, *p* = 0.007) and re-attendance within 30 days of discharge after admission (OR = 2.73, 95% CI 1.27–5.88, *p* = 0.01). Frailsafe screen results independently predict a range of outcomes amongst older ED attendees.

## 1. Introduction

Life expectancy in the United Kingdom has increased significantly for men and women and it is projected that the proportion of people aged 65 years and over will continue to increase to 23% of the total population by 2035 [1,2]. Advancing age is associated with frailty; between a quarter and a half of over-85-year-olds are thought to be frail, which brings with it an increased risk of developing disability, requirements for long-term care, and death [3]. As the population ages, there is a clear need for urgent care providers to develop services that can meet the projected rising demand for high-quality care of frail older people.

The Emergency Department (ED) is not traditionally seen as having a major role in managing older patients, with conventional models largely based on the rapid assessment and discharge of patients according to clinical severity and/or trauma management. However, people aged 65 and over account for one in every five English ED attendances [4], and 47% of patients who are admitted from the ED are aged 65 and older [5]. Older patients admitted to hospital are at significant risk of adverse outcomes, with longer admissions, high rates of functional and cognitive decline, greater risk of readmission and institutionalisation after discharge, and mortality [6,7]. Even amongst those who are discharged directly from the ED, there is a high rate of re-attendance, hospitalisation and mortality after discharge [8,9]. 

The British Geriatrics Society have undertaken work to facilitate quick and effective identification and management of older patients with frailty syndromes in acute hospital admissions, and pilot schemes are currently underway to validate a safety checklist, called Frailsafe, for potentially at risk older patients admitted to acute hospital settings [10]. This is not a frailty screening tool in itself, but a series of recommendations designed to improve the reliability of frail older people’s care in acute hospitals [10]. Its design encompasses rapid identification of at-risk older patients, via a three-point screening question, and subsequent recommendations for further assessments and actions via a frailty safety checklist. This study aims to evaluate whether the three-point Frailsafe screening questions, but not the subsequent safety checklist, have any predictive value for a range of adverse hospital outcomes when used in an ED setting.

## 2. Materials and Methods 

### 2.1. Design and Setting

This study took place in the ED of the University Hospital of Wales, an acute teaching hospital, which covers a population of 489,931 people, of whom 76,776 are over 65 years [11]. Data were retrospectively collected via a combination of case-note reviews and the use of hospital electronic databases.

### 2.2. Study Sample

Consecutive presentations for patients aged ≥75 years who presented to the ED during a two-week period in August 2015 were screened for eligibility. All unplanned self-presentations were reviewed and included in the study. Excluded cases were scheduled returners or patients referred in by a community practitioner, as it was not possible to differentiate between an acute presentation and follow-up appointments. Only the first presentations were included in the data analysis. Follow-up data was collected using the hospital electronic record system. Patients who were not a resident within the health board were excluded from follow-up after discharge, due to their data not being available. Permission to conduct the study was granted by the Cardiff and Vale University Health Board Research and Development Committee.

### 2.3. Data Collection

#### 2.3.1. Baseline Data

Patients were identified using the ED electronic patient registration database. Demographic information (age, sex) and destination on leaving the ED (admitted, discharged or died) were also collected. Case-notes were screened for the following information: clinical acuity on arrival (scored according to Manchester Triage Category (MTC) and/or National Early Warning Score (NEWS)), Charlson Comorbidity Index (CCI), polypharmacy (defined as being prescribed five or more medications) and cognitive impairment (recorded as present if the notes contained information pertaining to a known diagnosis or if the history alluded to confusion or disorientation, acute or chronic).

As a number of case-notes contained either MTC or NEWS, a Combined Acuity Score (CAS) was calculated, where acuity was classed as more severe (NEWS ≥3 or MTC category 1–3) or less severe (NEWS <2 or MTC category 4–5).

#### 2.3.2. Frailsafe Questions

Patients were classified as ‘Frailsafe positive’ if they scored one or more from the three Frailsafe screen criteria: does the patient have reduced mobility; is the patient confused and/or does the patient reside in a care home [10]. Each of the three factors has equal weighting. Discretion was used in recording a subject as Frailsafe positive if the presence of these factors was unlikely to be related to frailty according to clinical notes, as is recommended in use of the Frailsafe tool.

### 2.4. Outcomes

The primary outcome was mortality at 180 days. Secondary outcomes assessed between Frailsafe positive and Frailsafe negative patients were death at 30 and 120 days, a new care home admission at 180 days and a combined long-term outcome, which was defined as either a patient dying within 30, 120 or 180 days or being institutionalised at 180 days from presentation to the ED. The study also assessed Length of Stay (LOS) >14 and >28 days, inpatient mortality, new care home placement on discharge, 30-day re-attendance post discharge if admitted, a combined adverse outcome (any of: LOS >28 days, inpatient mortality, new care home on discharge or 30-day re-attendance) and re-attendance within 30, 120 and 180 days of immediate discharge from the ED.

### 2.5. Data Analysis

Data were analysed using the Statistical Package for the Social Sciences (SPSS), version 23. The Student’s t test and Mann–Whitney U test were used for parametric and non-parametric continuous data, respectively. Chi-squared testing was used to compare categorical data. 

Univariate logistic regression was performed for outcomes across the range of potential predictors (age, sex, Frailsafe status, CCI, polypharmacy and CAS).

Results were adjusted for confounding factors through a multivariate logistic regression model using the variables shown to be predictive in the univariate analysis. Results for the primary outcome of mortality at 180 days, plus mortality at 30 and 120 days were analysed for sensitivity, specificity and predictive power expressed as the area under the receiver operator curve (AUC), where the AUC >0.70 represents an acceptable discriminatory power, 0.60–0.69 and 0.50–0.59 represent a poor and very poor discriminatory power, respectively. 

## 3. Results

A total of 618 patients aged ≥75 presented to the ED during the study period. After excluding planned attendances and patients who re-presented, 468 were included. Complete data were available for destination from ED, length of hospital stay, inpatient mortality and new care home admission for all 468 patients. Follow-up data regarding re-attendance for patients discharged from ED, re-attendance within 30 days of discharge if admitted, mortality and new care home admission were available for 452 (96.6%) patients; follow-up data were not available for 16 patients who lived outside the health board catchment area.

Data were missing across a range of variables due to insufficient information recorded in the clinical notes. Typically, this was seen with minor complaints that did not require extensive clerking. Complete data sets were unavailable for the following variables: CCI, polypharmacy, cognitive impairment, CAS on arrival and Frailsafe screening questions score.

### 3.1. Baseline Characteristics of Frailsafe Screen Positive vs. Frailsafe Screen Negative Patients

Of the 468 case notes reviewed, 356 (76.1%) contained sufficient data to determine Frailsafe classification, of whom 194/356 (54.5%) were classed as Frailsafe screen positive. Table 1 summarises the baseline characteristics and outcomes of Frailsafe screen positive vs. Frailsafe screen negative patients.

There was no significant difference in admission rates for Frailsafe positive and Frailsafe negative patients (*p* = 0.06).

### 3.2. Outcomes for All Patients

The outcome data with univariate and multivariate analyses is summarised in Table 2. 

### 3.3. Primary Outcome

A positive Frailsafe screen was a predictor of death within 180 days, and remained so after adjustment for sex, co-morbidities and clinical acuity (Adjusted Odds Ratio (AOR) = 3.23, 95% CI 1.45–7.19, *p* = 0.004). The sensitivity of the Frailsafe screening questions in predicting the primary outcome was 70.0% (95% CI 55.4–82.1%), with a specificity of 46.9% (95% CI 41.1–52.8%).

The discriminatory power (AUC) of the Frailsafe screening questions at predicting mortality at 30, 120 and 180 days was 0.64 (95% CI 0.58–0.69), 0.57 (95% CI 0.52–0.63) and 0.59 (95% CI 0.512–0.63) respectively.

### 3.4. Secondary Outcomes

Frailsafe screen status did not predict 30 or 120-day mortality after ED attendance, but there was an increasing trend for mortality at 120 days for Frailsafe screen positive patients. Male sex, CCI and higher clinical acuity on admission were predictors of mortality at 120 days, of which male sex and CCI remained predictors after adjustment. 

A positive Frailsafe was an independent predictor of a new care home admission at 180 days (AOR = 8.95, 95% CI 2.01–39.83, *p* = 0.004). Being Frailsafe positive was a predictor of the combined long-term adverse outcome of either new institutionalisation or mortality within 180 days of ED attendance after adjustment for other predictors of the combined long-term adverse outcome (age, sex, CCI and CAS), (AOR = 3.55, 95% CI 1.64–7.67, *p* = 0.001). Male sex, co-morbidity score and higher clinical acuity at ED attendance all remained predictors of the combined long-term adverse outcome after adjustment.

### 3.5. Outcomes for Patients Admitted from ED

A positive Frailsafe was a predictor for lengths of stay of >14 and >28 days, but only remained significant after adjustment at >28 days (AOR 3.42, 95% CI 1.41–8.31, *p* = 0.007). 

Age was also a predictor of prolonged LOS of both >14 and >28 days and remained significant after adjustment at >14 days only (AOR = 1.06, 95% CI 1.01–1.13, *p* = 0.027). A positive Frailsafe was an independent predictor for re-attendance within 30 days of discharge after an admission to hospital following the index ED visit (OR = 2.73, 95% CI 1.27–5.88, *p* = 0.01).

Frailsafe screen status did not predict inpatient mortality (OR = 3.00, 95% CI 0.82–10.99, *p* = 0.097); male sex was the only independent predictor of inpatient mortality following admission from ED (OR = 2.76, 95% CI 1.15–6.60, *p* = 0.023). A positive Frailsafe did not predict new care home admission on discharge from hospital (OR = 1.13, 95% CI 0.18–6.92, *p* = 0.89).

Being Frailsafe positive was an independent predictor of suffering any admission-related adverse outcome when compared to Frailsafe negative patients (OR = 2.67, 95% CI 1.42–4.99, *p* = 0.002).

### 3.6. ED Re-Attendance

Frailsafe screen status did not predict re-attendance at any time point for patients who were discharged directly from the ED. Higher clinical acuity at initial attendance was an independent predictor after adjustment at both 120 days (AOR = 2.07, 95% CI 1.04–4.11, *p* = 0.039) and 180 days (AOR = 2.90, 95% CI 1.46–5.75, *p* = 0.002), whilst age remained significant after adjustment at 180 days only (AOR = 1.07, 95% CI 1.01–1.14, *p* = 0.023).

## 4. Discussion

This is the first study to our knowledge that describes the use of the Frailsafe screening questions in the ED and has shown it to have an association with a range of adverse outcomes. These associations were seen for mortality, admission to a care home and longer length of hospital stay; however, the comparatively wide confidence intervals imply caution when interpreting the results.

A positive Frailsafe predicted increased odds of mortality, institutionalisation and a combined long-term outcome (mortality or institutionalisation) within 180 days of presentation to the ED. For those admitted it was an independent predictor of prolonged LOS of >28 days, 30-day re-attendance post discharge and the combined admission-related adverse outcome. These results indicate that the Frailsafe questions may indeed have a realistic role in highlighting individuals at risk of adverse hospital outcomes when used in an ED setting. 

Frailsafe status failed to predict inpatient mortality, new institutionalisation on discharge, and early mortality (within 30 days), which may reflect the relatively low numbers of events.

Although more Frailsafe positive patients had re-attended at final follow-up, this was not significant and Frailsafe outcome was not predictive of ED return amongst those who were discharged. Predicting ED re-attendance amongst high-risk older patients has been the subject of wider research, with limited consensus on an appropriate tool to use in this distinct group. In a study of 851 older American ED patients, frailty defined by a deficit accumulation index was a predictor of adverse outcomes (hospitalisation, nursing home admission or death) within 30 days of an ED visit for the most frail vs. the least frail participants, but no association was identified between degree of frailty and repeat ED attendance within 30 days [12]. The correct tool to predict ED return in this cohort remains unclear, and our research echoes this.

Designing a suitable tool to use in the ED setting to accurately risk-stratify older patients, with adequate power to identify high risk individuals, with simplicity and ease of use is challenging. The Identification of Seniors at Risk (ISAR) questionnaire was designed for use in an ED setting and at a cut-off score of ≥2 has been shown to have modest predictive accuracy in the ED for a range of outcomes (including functional decline, future hospitalisation and mortality) [13]. A meta-analysis of pooled data from 32 studies found the ISAR to have a sensitivity of 87% and specificity of 35% for predicting mortality within six months of an ED visit [13]; this compares with a sensitivity and specificity for the Frailsafe questions of 70% and 47% respectively, whilst both have a similar discriminatory power (AUC for ISAR 0.57 vs. AUC for Frailsafe questions 0.59). However, the ISAR, although relatively simple and quick to complete, requires the patient or a proxy to respond to a list of questions. Thus, frail patients unable to respond due to cognitive impairments may be excluded. The Frailsafe criteria avoids this issue and could be a practical alternative to ISAR. 

The Clinical Frailty Scale (CFS), which provides descriptive information regarding an individual’s degree of frailty, demonstrated a good ability to predict inpatient death (AUC 0.72 (95% CI 0.69–0.75)) and transfer to geriatric medicine beds (AUC 0.68 (95% CI 0.66–0.71)) when used to screen emergency admissions in a large UK hospital [14]. However, it is worth noting that, of the eligible patients in this study, 23.5% did not undergo CFS screening—many of whom either presented with greater illness severity or were admitted to areas with anticipated shorter lengths of stay. Using a pragmatic, simple tool such as the Frailsafe tool may help increase uptake of screening for patients at risk of adverse outcomes in such settings, subsequently allowing more detailed frailty assessment to be completed for the most appropriate individuals. In a survey of ED clinicians, the majority felt that frailty identification should take less than one minute, and so it is important that any tool used in this setting should be both straightforward and quick to administer [15], the simplicity of the Frailsafe means it should easily fulfil this criteria. It must, however, be acknowledged that Frailsafe scores were only obtained for 76.1% of those eligible in this study due to insufficient data being available in written case notes. Further data collection and study with a dedicated Frailsafe checklist is warranted prior to recommending its implementation as a quick screening tool in the ED.

A key strength of this observational study is that Frailsafe screening was used in an unselected ED population. This allows a more realistic picture of the prevalence and outcomes for at risk older people in the ED. It remains unclear what is the most appropriate tool to use to identify older patients at risk of adverse hospital outcomes in this setting, so data that reflects a true picture of patients who require ED care is vital to allow the development of new service models. 

In this study, a reasonable proportion of Frailsafe negative participants also suffered adverse outcomes; this likely, in part, reflects the use of case-note reviews to accurately detect the presence of Frailsafe criteria, as some patients may have been misclassified due to a lack of complete or accurate documentation. Further study based on the prospective use of the tool would allow more precise scoring and enable a more accurate exploration of the association between the Frailsafe screen and adverse hospital outcomes.

This study lacks data on quality and patient-related outcomes and instead focuses on service outcomes. These are important, as planning new services requires robust evidence of potential cost savings in addition to patient benefits; however, future work would ideally include a wider range of measurements.

Despite a reasonable sample size, the numbers for some outcomes were small and, subsequently, the confidence intervals were wide for some variables. The collection of data from a larger cohort would add power and likely yield greater results. 

This is a single-site study which took place over a relatively short time period, during the summer months, and may simply be a reflection of the local population and service operations and may not be widely generalisable. However, the University Hospital of Wales is a fairly representative UK teaching hospital with a range of socioeconomic patients from both rural and urban areas. 

A positive Frailsafe was associated with a greater likelihood of experiencing a range of adverse outcomes, both in the short term for those who were admitted and at the end of the six-month follow-up for all patients. The ability of the Frailsafe screening process to predict adverse outcomes, as identified in this study, combined with its relative simplicity and ease of use, suggest it could be a useful tool to facilitate the identification of at-risk older patients in hospital emergency departments.

## Figures and Tables

**Table 1:** Baseline characteristics for all patients and for patients scored according to the Frailsafe screening questions (*p* value for comparison between Frailsafe screen positive and Frailsafe screen negative groups). (Charlson Comorbidity Index (CCI); Early Warning Score (EWS); Combined Acuity Score (CAS)).

	All Patients	Frailsafe Positive N = 194	Frailsafe Negative N = 162	*p* Value
Age [mean (SD)] N = 468	83.9 (5.7)	85.8 (5.8)	82.2 (5.4)	<0.001
Sex [Female, N(%)] N = 468	270 (57.7)	123 (63.4)	89 (54.9)	0.105
CCI [mean (SD)] N = 300	1.8 (2.3)	2.0 (2.2)	1.6 (2.4)	0.800
Polypharmacy [n(%)] N = 306	185 (60.5)	102 (52.6)	69 (42.6)	0.009
Cognitive Impairment [n(%)] N = 342	92 (25.8)	92 (47.4%)		
EWS on admission [mean(SD)] N = 342	0.8 (3.1)	0.8 (1.4)	0.7 (1.3)	0.922
Manchester Triage Category [mean(SD)] N = 223	3.13 (0.79)	3.08 (0.75)	3.10 (0.81)	0.025
CAS – High Acuity (%) N = 371	182 (49.1)	95 (49.0)	65 (40.1)	0.255
Admitted from ED [N(%)]	253	116 (59.8%)	81 (50%)	0.064

**Table 2:** Univariate and multivariate logistic regression analyses for the presence of a positive Frailsafe screen and any other variables shown to be a positive predictor for each outcome (odds ratio (OR); 95% confidence interval (95% CI); adjusted odds ratio (AOR); Charlson Comorbidity Index (CCI); Combined Acuity Score (CAS); Length of Stay (LOS)).

	OR	95% CI	*p*	AOR	95% CI	*p*
**30 Day Mortality**						
Frailsafe Positive	0.31	0.06–1.64	0.168			
**120 Day Mortality**						
Frailsafe Positive	1.85	0.88–3.92	0.106			
Sex	2.57	1.42–4.64	**0.002**	3.06	1.31–7.12	**0.009**
CCI	1.19	1.05–1.37	**0.009**	1.16	1.00–1.33	**0.043**
CAS	2.48	1.20–5.10	**0.014**	1.86	0.79–4.36	0.154
**180 Day Mortality**						
Frailsafe Positive	2.06	1.08–3.94	**0.028**	3.23	1.45–7.19	**0.004**
Sex	2.07	1.25–3.45	**0.005**	2.93	1.41–6.11	**0.004**
CCI	1.23	1.09–1.38	**0.001**	1.23	1.09–1.45	**0.002**
CAS	2.74	1.48–5.07	**0.001**	2.45	1.14–5.28	**0.021**
**New Care Home at 180 Days**						
Frailsafe Positive	10.84	2.50–47.00	**0.001**	8.95	2.01–39.83	**0.004**
Age	1.12	1.04–1.20	**0.002**	1.05	0.98–1.13	0.18
**Combined Long Term Outcome**						
Frailsafe Positive	2.99	1.65–5.44	**<0.001**	3.55	1.64–7.67	**0.001**
Age	1.06	1.01–1.10	**0.009**	1.04	0.98–1.10	0.205
Sex	1.95	1.23–3.08	**0.005**	3.31	1.67–6.53	**0.001**
CCI	1.18	1.06–1.32	**0.003**	1.23	1.06–1.41	**0.005**
CAS	2.62	1.52–4.51	**<0.001**	2.16	1.09–4.32	**0.028**
**LOS >14 Days**						
Frailsafe Positive	2.08	1.08–4.02	**0.029**	1.89	0.97–3.70	0.062
Age	1.06	1.01–1.12	**0.013**	1.06	1.01–1.13	**0.027**
**LOS >28 Days**						
Frailsafe Positive	3.72	1.54–8.98	**0.003**	3.42	1.41–8.31	**0.007**
Age	1.08	1.02–1.14	**0.014**	1.06	1.00–1.13	0.073
**Inpatient Mortality**						
Frailsafe Positive	3.00	0.82–10.99	0.097			
Sex	2.76	1.15–6.60	**0.023**			
**New Care Home On Discharge**						
Frailsafe Positive	1.13	0.18–6.92	0.896			
**30 Day Re-Attendance Post Hospital Discharge**						
Frailsafe Positive	2.73	1.27–5.88	**0.010**			
**Combined Admission Related Adverse Outcomes**						
Frailsafe Positive	2.67	1.42–4.99	**0.002**			
**Re-Attendance within 30 Days to ED**						
Frailsafe Positive	0.82	0.34–1.96	0.657			
Age	1.07	1.02–1.13	0.010	1.05	0.99–1.11	**0.140**
CAS	2.10	1.06–4.16	0.033			
**Re-Attendance within 120 Days to ED**						
Frailsafe Positive	1.51	0.77–2.96	0.233			
Age	1.07	1.02–1.13	**0.01**	1.05	0.99–1.11	0.140
CAS	2.10	1.06–4.16	**0.033**	2.07	1.04–4.11	**0.039**
**Re-Attendance within 180 days**						
Frailsafe Positive	1.31	0.69–2.51	0.410			
Age	1.09	1.03–1.15	**0.001**	1.07	1.01–1.14	0.023
CAS	2.91	1.49–5.71	**0.002**	2.90	1.46–5.75	**0.002**

Bold means statistically significant.
